# Wildfires lead to decreased carbon and increased nitrogen concentrations in upland arctic streams

**DOI:** 10.1038/s41598-020-65520-0

**Published:** 2020-05-26

**Authors:** B. M. Rodríguez-Cardona, A. A. Coble, A. S. Wymore, R. Kolosov, D. C. Podgorski, P. Zito, R. G. M. Spencer, A. S. Prokushkin, W. H. McDowell

**Affiliations:** 1 Department of Natural Resources and the Environment, University of New Hampshire, Durham, NH USA; 2 National Council for Air and Stream Improvement, Inc., Corvallis, OR USA; 3 V.N. Sukachev Institute of Forest SB RAS, Krasnoyarsk, Russia; 4 Pontchartrain Institute for Environmental Sciences, Department of Chemistry, University of New Orleans, New Orleans, LA USA; 5 Department of Earth, Ocean and Atmospheric Sciences, Florida State University, Tallahassee, FL USA; 6 Present Address: Département des Sciences Biologiques, Université du Québec à Montréal, Montréal, Québec Canada

**Keywords:** Biogeochemistry, Limnology

## Abstract

The Central Siberian Plateau is undergoing rapid climate change that has resulted in increased frequency of forest fires and subsequent alteration of watershed carbon and nutrient dynamics. Across a watershed chronosequence (3 to >100 years since wildfire) we quantified the effects of fire on quantity and composition of dissolved organic matter (DOM), stream water nutrient concentrations, as well as in-stream nutrient uptake. Wildfires increased concentrations of nitrate for a decade, while decreasing concentrations of dissolved organic carbon and nitrogen (DOC and DON) and aliphatic DOM contribution for five decades. These post-wildfire changes in stream DOM result in lower uptake efficiency of in-stream nitrate in recently burned watersheds. Nitrate uptake (as uptake velocity) is strongly dependent on DOM composition (e.g. polyphenolics), ambient dissolved inorganic nitrogen (DIN), and DOC to DIN ratios. Our observations and experiments suggest that a decade-long pulse of inorganic nitrogen and a reduction of DOC export occur following wildfires in streams draining the Central Siberian Plateau. Increased fire frequency in the region is thus likely to both decrease DOM and increase nitrate delivery to the main stem Yenisei River, and ultimately the Arctic Ocean, in the coming decades.

## Introduction

Arctic soils store large amounts of organic matter (OM) and nutrients in permafrost^1,2^. When permafrost is degraded, interactions between nutrient availability and OM composition will play an important role in determining the net effects on delivery of dissolved organic carbon (DOC) and inorganic nutrients from upland landscapes to major arctic rivers and ultimately the Arctic Ocean^2,3^. Predicted and recently observed changes in the concentration and export of OM and nutrients vary geographically across the Arctic and as a function of permafrost structure, underlying parent material, vegetation, and disturbance^3,4^. Boreal forests have been burning with greater frequency due to longer growing seasons, warmer temperatures, and changing weather patterns^5–7^, thereby adding additional uncertainty to projections of how these ecosystems will respond to climate change. The response of arctic watersheds varies dramatically among regions, with both increases and decreases in DOC concentrations and inorganic nutrients following wildfire, and differing return intervals to baseline conditions^8–13^. Although various studies have documented the effects of fire on stream chemistry, few have evaluated how these changes will impact the processing and export of nutrients from Arctic fluvial systems. Arctic rivers mobilize large quantities of organic matter and nutrients to the Arctic Ocean^14^ which may be altered due to increasing permafrost thaw and wildfire frequency. Watersheds in the Central Siberian Plateau, a major component (2.5 million km^2^) of the Yenisei River Basin, provide a platform for understanding the long-term resilience and recovery of stream chemistry following fire in taiga landscapes. Streams of the Central Siberian Plateau drain watersheds with extensive larch forest (*Larix gmelinii)* growing on continuous permafrost (300–500 m deep) underlain by unglaciated basalt deposits^15,16^.

Uptake and transformation of nutrients and OM by stream channel processes can result in losses of C and N through mineralization and denitrification, as well as alteration of the timing of delivery of DOC and nitrate (NO_3_^-^) to downstream ecosystems^17–20^. Uptake rates (usually expressed as uptake velocity, V_f_) are commonly used to quantify nutrient demand and the efficiency with which in-stream biota remove and process nutrients at a given concentration^21,22^. Uptake velocity is typically determined via *in-situ* nutrient manipulations since uptake is difficult to infer from downstream changes in the ambient pool of nutrients. Generally, as nutrient concentrations increase, uptake efficiency of the microbial community declines, resulting in a larger fraction of nutrients exported downstream^17,23^. Uptake dynamics have not been evaluated extensively in arctic fluvial systems affected by wildfires^10^.

**Figure 1:**
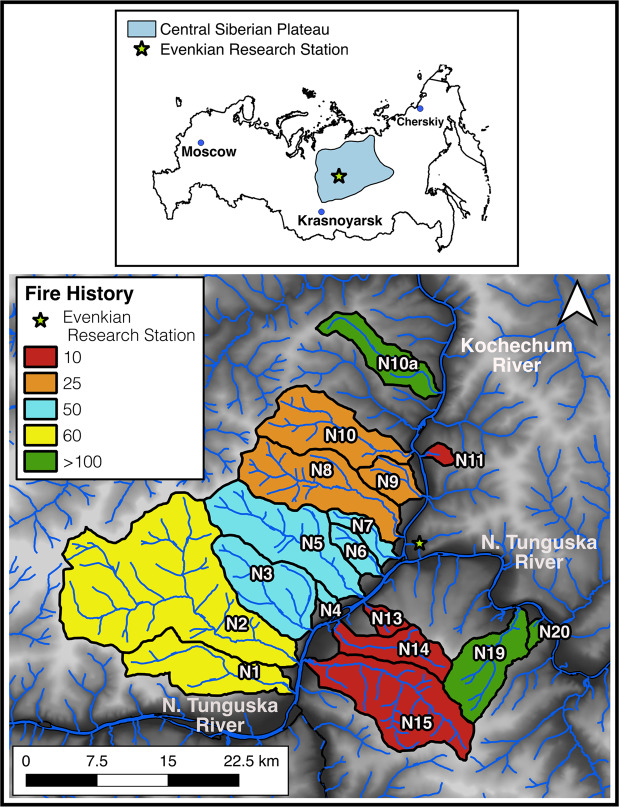
Map of sub-watersheds sampled in the Central Siberian Plateau, blue region shown in insert of Russia. Each watershed is colored with its respective fire history between 3 and >100 years since the last fire, draining into the Kochechum River and Nizhnyaya Tunguska River which are part of the greater Yenisei River Basin. On the fire history key, 10 represents watersheds burned between 3 and 7 years ago, 25 are watersheds that burned between 18 and 25 years ago, 50 represents watersheds between 51 and 57 years ago, 60 represents watersheds burned between 66 and 71years ago, and >100 are watersheds that burned up to 122 years ago. See Supplementary Table 1 for further details on individual watersheds.

Here we examine how years since the last fire affect nutrient concentrations and the quantity and composition of dissolved organic matter (DOM) entering fluvial networks using a space-for-time approach spanning a 100-year well-constrained wildfire chronosequence across 17 watersheds (Fig. 1) (Supplementary Table 1) in the Central Siberian Plateau^16^. We also determined uptake rates of inorganic N in a subset of these watersheds with individual nutrient pulse additions^24^ of nitrate (NO_3_^−^) and ammonium (NH_4_^+^) to determine how fire influences the processing and export of nutrients in arctic fluvial systems. We assess changes to the composition of DOM via optical properties and Fourier transformed ion cyclotron resonance mass spectrometry (FT-ICR MS) to provide an ultra-high resolution assessment of molecular formulae for thousands of individual DOM molecules (>10,000)^25^ in multiple water samples from each stream. Samples were taken in multiple years during June (freshet) and July (summer low flow).

**Figure 2:**
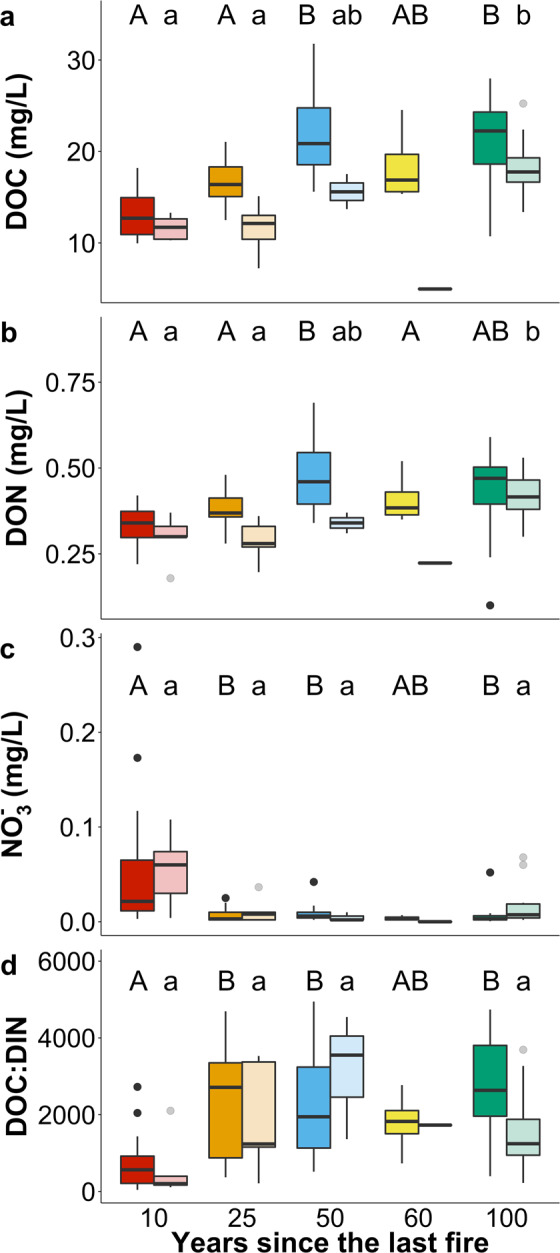
DOM, nutrient concentrations, and stoichiometric ratios across the burn gradient. Boxplot panels represent (**A**) DOC, (**B**) DON, (**C**) NO_3_^–^N concentrations, and (**D**) molar DOC:DIN ratios, across 17 streams sampled 2011, 2013, and 2016–2017 during June and July across the burn gradient. The paired boxplots correspond to June (dark shade) and July (lighter shade). Boxes represent interquartile range with the median value as the bold line, whiskers represent 1.5 interquartile range, and points are possible outliers. Letters denote significant differences (α = 0.05) where uppercase correspond to June and lowercase to July. Note that July data in 60 years since the last fire was excluded from statistical analysis due to low n. Significant differences were tested using ANOVA for the parametric variables and Kruskal-Wallis test for nonparametric variables. Statistics for DOC June p = 1.8 × 10^−8^ and July p = 8.3 × 10^−4^; DON June p = 0.0002 and July p = 0.005; DIN June p = 0.008 and July p = 0.04; DOC:DIN June p = 0.0008 and July p = 0.06. Respective n-values across the burn gradient for June: 16, 16,19, 4, 10; July: 5, 5, 3, 1, 10.

Fires altered concentrations of DOC, dissolved organic nitrogen (DON), and nutrients in streams of the Central Siberian Plateau. Concentrations of DOC (Fig. 2A; p = 5.87 × 10^−9^, F = 14.33, df=4) and DON (Fig. 2B; p = 2.78 × 10^−5^, F = 7.61, df=4) both increased with longer time since the last fire, with patterns consistent in June and July. Similar responses between June and July reflect consistency across changing flow regimes shifting from run-off dominated spring freshet in early June, and flows in July that are more representative of low-flow conditions typical of summer. In recently burned sites DOM inputs to the stream were likely reduced due to the combustion of the surface litter and organic layer, which reduces soil standing stocks of C and production of DOM from soil OM^7^. Post-fire declines in stream water DOC and DON concentrations can also be attributed to deepening of the active layer, which exposes mineral soil^2,3^ leading to increased adsorption of DOM^3,15,26^. Changes to concentrations of DOC and DON across the chronosequence indicate an ecosystem recovery time of approximately 50 years post wildfire disturbance, during which time the vegetation (moss, lichens, trees) undergoes regrowth re-establishing OM inputs into the soil. DOC concentrations in central Siberia exhibit a positive relationship with forest productivity and can serve as a proxy for forest C stocks^15,16^. The stabilization of DOC and DON concentrations after 50 years since the last fire suggests the reestablishment of an equilibrium as described by resilience theory and the ecosystem succession and nutrient retention hypothesis^27,28^.

Stream dissolved inorganic nitrogen (DIN) responded differently to forest fires than DOM. NO_3_^−^ concentrations were highest in the most recently burned sites and decreased with increasing recovery time (Fig. 2C, p«0.05); consistent responses to fire were observed in both June and July. Multiple mechanisms have been proposed to explain the elevated NO_3_^−^ concentrations often observed in recently burned watersheds on permafrost, such as an increase in soil aeration from improved drainage after fire leading to declines in soil denitrification^9^, increases in mineralization-nitrification of organic nitrogenous compounds^29^ and decreased nutrient uptake due to the reduction in biomass^30,31^. In contrast to DOC and DON, concentrations of NO_3_^−^ demonstrate a shorter recovery time, returning to their pre-disturbance concentrations approximately at 10 years. In larch forests post-fire, growth of the moss layer and OM storage is very slow^32^ but there is a greater demand for inorganic N from growing shrubs and larch seedlings, which require fire to propagate^32,33^. This relatively short recovery of NO_3_^−^ in the Central Siberian Plateau is consistent with earlier studies, which have found recovery intervals of 5–6 years after fire for inorganic N in other boreal streams^30^. Since the recovery of DOC and DON concentrations lags behind that of NO_3_^−^, it appears that the processes which produce, and export DOM serve as the rate-limiting step in determining the resilience and stability of heterotrophic processes in these watersheds. As a result, these Central Siberian Plateau streams at 10 years remain in a state of relatively higher energy limitation compared to their pre-disturbance steady-state (i.e. watersheds that were burned over 100 years ago) as described by molar DOC:DIN ratios (Fig. 2D; p = 9.78 × 10^−5^). In the study streams, DIN is mostly composed of NO_3_^−^ where ammonium (NH_4_^+^) is often at or below detection limit (~5 µg/L as N). During the post-fire interval when DIN is elevated and DOC is reduced, this balance between energy and nutrient demand (as measured by stoichiometry) is re-established after approximately ten years reflecting a system that returns to a state of inorganic-N limitation and high DOM availability. During this initial recovery period when DON concentrations are relatively low, the predominant in-stream nutrient source may shift from organic to inorganic forms of N. There was no statistically significant difference in ammonium and phosphate concentrations across the burn gradient (Supplementary Table 2).

**Figure 3:**
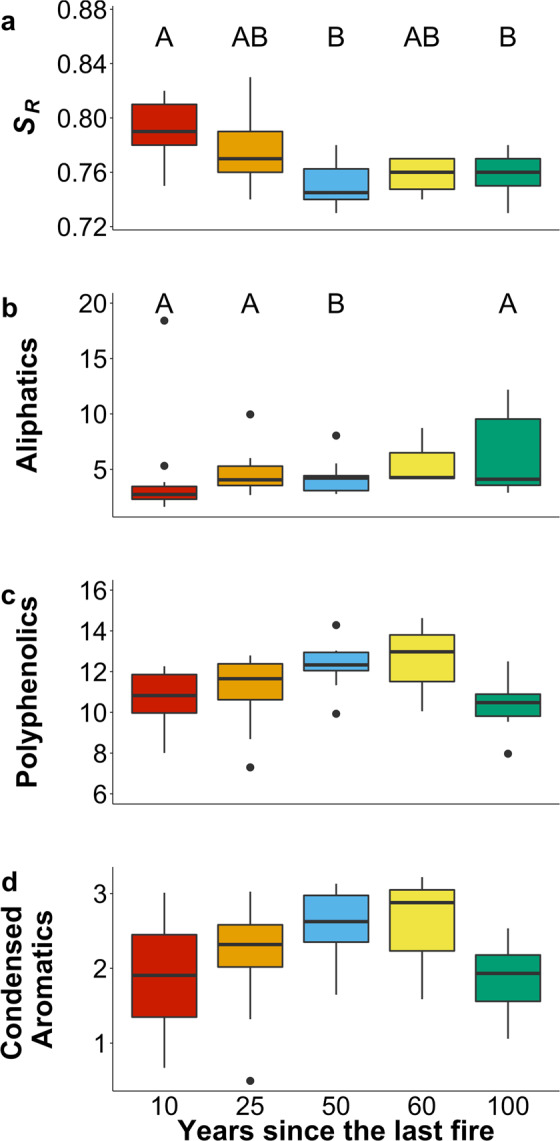
DOM composition of streams across the burn gradient. Boxplots represent (**A**) slope ratios (*S*_*R*_) from June 2013 and 2016, relative abundance of (**B**) aliphatic compounds including N-containing aliphatics, (**C**) polyphenolic compounds, and (**D**) condensed aromatic compounds. Streams burned 60 years ago for FT-ICR MS were excluded from statistical analyses due to low n. Boxes represent interquartile range with the median value as the bold line, whiskers represent 1.5 interquartile range, and points are possible outliers. Uppercase letters denote significant differences (α = 0.05). Only data from June 2016 for FT-ICR MS compound groups and June 2013 and 2016 for *S*_*R*_. Significant differences were tested using Kruskal-Wallis test for nonparametric variables. P-value for *S*_*R*_ p = 0.008, aliphatics 0.047, polyphenolics p = 0.07, and condensed aromatics p = 0.18. Respective n-values across the burn gradient *S*_*R*_: 11, 9, 12, 4, 7; FT ICR: 11, 8, 9, 2, 6.

The effects of fire on landscape recovery are also reflected in DOM composition. As surface flow paths change with landscape recovery, exported OM shifts in quantity, composition, and bioavailability from microbial-derived low molecular weight DOM from deep soils to less processed and more aromatic DOM derived from surface flow paths^34^. This is reflected in the spectral slope ratios (*S*_*R*_) shifting from low molecular weight to higher molecular weight values with increasing time since fire (Fig. 3A, p = 0.002). Aliphatic compounds (including N-containing aliphatics) increased with increasing recovery time (Fig. 3B; p = 0.04, F = 2.96, df=3) while polyphenolic and condensed aromatic compounds decreased after 60 years since the last fire although the change was not statistically significant (Fig. 3C p = 0.07, F = 2.49, df=3; Fig. 3D p = 0.18, F = 1.72, df=3). With the prolonged absence of fire in boreal forests, decomposition rates decline thus promoting greater storage of OM in soils^35^, a change in watershed C dynamics that is reflected in the increase of aliphatic compounds. Condensed aromatic compounds, including poly-condensed aromatic compounds derived from charred material that are known as black carbon^25,36^ were relatively low across our study watersheds, similar to others that have also found no clear relationship between black carbon in streams and fire history^37^. DOM composition as assessed by ultrahigh-resolution mass spectrometry reflects the recovery of the adjacent landscape, patterns that are not evident using DOM optical properties only (Supplementary Table 3) or the four component PARAFAC model that describes mostly humic-like DOM (Supplementary Table 4).

Biogeochemical regimes in stream ecosystems reflect multiple physical, chemical, and biological processes occurring at the watershed scale as well as underlying geology and climate. Wildfire disturbance interacts with each of these drivers to determine stream chemistry and microbial biogeochemical processes. To test hypotheses regarding the potential role of other landscape scale drivers we used a backward stepwise regression to relate stream chemistry and DOM composition to watershed characteristics (area, slope, elevation, aspect, years since the last fire, and season as described by either freshet period or low discharge). Across the different models, years since the last fire and season consistently were the most significant predictors of DOC, DON, and DIN concentrations, DOC:DIN ratios, and DOM composition (Supplementary Table 5). Although season was a strong predictor of solute concentrations, the patterns across the chronosequence remain similar despite the differences in concentrations. Others have also found that time since wildfire disturbance also emerges as a strong predictor variable of stream chemistry even when additional landscape variables are included^38^.

**Figure 4:**
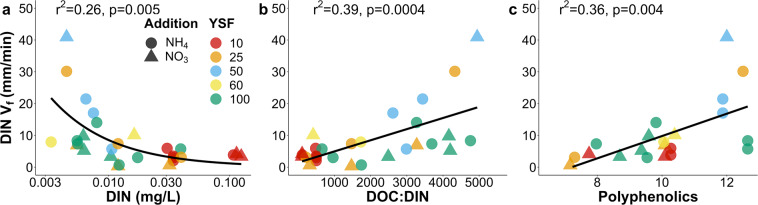
Drivers of DIN uptake velocity. Uptake velocity (V_f_) of NH_4_^+^ (circles) and NO_3_^−^ (triangles) grouped as DIN V_f_ from streams across the burn gradient (10 red, 25 orange, 50 blue, 60 yellow, and>100 green) related to (**A**) ambient DIN concentration, (**B**) molar DOC:DIN ratios, and (**C**) relative abundance of polyphenolics. V_f_ values here are from 2016 through 2018. The n-values for NH_4_^+ ^V_f_ is 18 and NO_3_¯ V_f_ is 10.

The response in stream chemistry due to fires influences the processing and export of DIN. Across all watersheds, DIN uptake velocities (V_f_) correlated negatively with DIN concentrations (Fig. 4A, r^2^ = 0.26, p = 0.005). This pattern has been observed in numerous studies in a variety of biomes, suggesting that the efficiency with which the microbial community removes DIN decreases with increasing DIN concentrations^17,23,39^. The changes in concentrations of DOC due to fires also influence DIN processing where streams with greater DOC:DIN molar ratios supported the greatest DIN removal (Fig. 4B r^2^ = 0.39, p = 0.0004). Relationships between uptake and molar ratios^19,20^ demonstrate the important role of DOM in DIN processing and indicate that the balance and interactions between DOM and DIN can influence the resilience of heterotrophic processes. Moreover, DOM compound groups such as polyphenolics, can enhance DIN removal (Fig. 4D, r^2^ = 0.36, p = 0.004); increasing relative abundance of polyphenolics leads to higher DIN uptake velocities. The positive relationships between DIN uptake and polyphenolics (i.e. vascular plant derived DOM^40^) suggests that uptake will be greatest in watersheds that have longer recovery times since the last fires, where inputs of DOM sources from terrestrial plants can be elevated. In addition, polyphenolics which have typically been thought to be aromatic and less bioavailable may be important energy sources in potentially energy limited streams (low primary productivity) despite high ambient DOM^41^. Alternatively, greater availability of DIN could have enhanced DOM decomposition by relieving any DIN limitations that inhibit the breakdown of aromatic molecules like polyphenolics. Although nutrient pulse additions do not allow us to determine what specific processes are responsible for DIN removal from the water column, the data demonstrate a close relationship between DOM and DIN uptake, supporting the idea that these patterns are due to heterotrophic or assimilative processes that require an energy source such as DOM. With shorter fire return intervals, elevated DIN concentrations, and declines in both DOM quantity and DOC:DIN ratios we can expect greater export of DIN as the strength of watersheds as sinks is greatly reduced leading to larger amounts of nutrients reaching larger rivers and ultimately the Arctic Ocean. DOC:DIN ratios provide a valuable integrator of energy versus nutrient limitation in stream ecosystems, in which energy limitation (low DOC:DIN ratios) or nutrient limitation (high DOC:DIN ratios) can be important in determining the delivery of nitrogen and OM to downstream ecosystems^19,20^. Downstream exports of inorganic N will likely persist for several years to a decade post fire, until DOM inputs from the watershed are re-established.

Across arctic watersheds, the response of stream chemistry to biomass burning is not consistent. Watershed recovery following wildfire across arctic watersheds appears to be a function of many variables, including composition of vegetation and rates of regrowth, nature and extent of OM consumed by fire, and the effects of both of these on active layer depth^3,7,9,12,33^. Although our study focuses on streams within the Central Siberian Plateau, the results presented here can be applied to a wide array of systems in northern high-latitude and boreal regions. This will better suit predictive models of how Arctic watersheds respond to fire disturbance across the pan-Arctic. The majority of previous studies include only the assessment of stream chemistry before and after a fire^8,9,13^, making predictions of long-term recovery and resilience difficult to evaluate and estimate. Our study addresses this challenge by taking advantage of a long chronosequence in a space-for-time substitution and combining observational and experimental data to better understand the response of watersheds to major landscape disturbances. Differences in the response to disturbance are likely driven by the role that permafrost structure (i.e. mineral soil layer vs. deep soil OM) and its continuity, hydrology, and vegetation communities (i.e. tundra vs. taiga) play in regulating elemental fluxes and the balance of energy and nutrient demand in arctic fluvial networks. A better understanding of the effects of large-scale perturbations like fires is important to develop a greater mechanistic understanding of how site conditions drive variability in responses, exports, and resiliency to increasing alteration of arctic landscapes.

## Methods

### Grab samples across chronosequence

We collected stream water samples in June and July from 2016 to 2018, but data from 2011^11^ and 2013^10^ are included in the main body of text. Samples were filtered through pre-combusted Whatman GF/F glass fiber filters and collected in acid washed high density poly-ethylene bottles (DOC, TDN, and nutrients), pre-combusted amber glass vials (optical DOM metrics), and polycarbonate bottles (FT-ICR MS) and rinsed 3 times in the field. Samples were kept frozen except for DOM optical samples which were kept at 4 °C; all samples were transported and analyzed at the Water Quality Analysis Laboratory at the University of New Hampshire, except samples collected for FT-ICR MS which were analyzed at the National High Magnetic Field Laboratory at Florida State University.

### Nutrient pulse additions

Short term nutrient pulse additions were performed at several sites across the fire chronosequence in June 2016, June and July 2017, and July 2018. Amendments consisted of NO_3_^−^ as NaNO_3_, and NH_4_^+^ as (NH_4_)_2_SO_4_, with and without PO_4_^3+^ as KH_2_PO_4_ but we report NH_4_^+^ uptake from both as PO_4_^3+^ had no influence on NH_4_^+^ uptake metrics. Amendments were added along with NaCl as a conservative tracer mixed with stream water where we increased background nutrient and Cl concentrations 2–3X above background. Experimental reach lengths ranged between 15 m to 200 m (see Supplementary Table 6 for more details on individual additions) and were selected to exclude large pools, tributaries, or other overland flows. The amendments were released at the top of the experimental reach and samples were collected at a fixed point at the bottom of the experimental reach throughout the break through curve (BTC), where between 20 and 40 samples were collected per experiment, depending on flow conditions and changes in specific conductance monitored with a ﻿HANNA HI 991300 (HANNA Instruments, Woonsocket, RI, US). ﻿Background samples were collected as described above in duplicates before every experiment at the top and bottom of the experimental reach.

Calculations of uptake metrics follow the break through curve integration method^24^ where uptake length (S_w_) was determined as the negative inverse of the ratio between the natural log of the fraction of background corrected nutrient and Cl retrieved and the reach length distance. Uptake velocity (V_f_) was determined as:

1 $${V}_{f}=\frac{\frac{Q}{w}}{{S}_{w}}$$

where Q is discharge, *w* stream width, and S_w_ the uptake length. Uptake velocity is the best metric to compare across sites and dates as it is normalized for stream depth and velocity^22–24^. Uptake velocity is reported in terms of DIN (combining NO_3_^−^ and NH_4_^+^ V_f_) due to low statistical power if reported individually. Discharge was determined using the dilution gaging method^42^ using NaCl dissolved in stream water and added to the stream as an instantaneous slug addition and conductivity was logged every 5 seconds using a HOBO conductivity data logger (Onset, Bourne, MA).

### Water chemistry

All samples were analyzed for DOC and total dissolved nitrogen (TDN) using high temperature catalytic oxidation with a Shimadzu TOC-V CPH/TNM. Nitrate (NO_3_^−^) was analyzed with ion chromatography using an Anion/Cations Dionex ICS-1000 with AS40 autosampler. Ammonium (NH_4_^+^) was measured using a SmartChem 200 discrete automated colorimetric analyzer using the alkaline phenate standard method. Phosphate (PO_4_^3−^) was analyzed as soluble reactive phosphorus by discrete colorimetric analysis on a Seal AQ2 (Seal Analytical) by the ascorbic molybdenum blue method (EPA 365.1). DON was determined arithmetically by subtracting DIN (NO_3_^−^ + NH_4_^+^) concentrations from TDN. Samples below detection limit were substituted with half the detection limit; method detection limit for NO_3_^−^ (4 µg/L as N), NH_4_^+^ (5 µg/L as N), PO_4_^3−^ (1 µg/L as P), DOC (0.05 mg/L), TDN (0.035 mg/L). Stream chemistry data was also obtained from the same streams as this study during June 2011^11^ and 2013^10^. Those samples were also collected and analyzed as described here.

### Optical properties

All grab samples across chronosequence from July 2013, June 2016, and July 2018 and background nutrient pulse samples from June 2016 and July 2018 were analyzed for DOM optical properties. UV absorbance was measured with a Shimadzu photo diode array detector with HPLC at 1 nm intervals between 200 to 700 nm. Specific UV absorbance at 254 nm (SUVA_254_) was determined by dividing the UV absorbance at 254 nm by DOC concentration, where SUVA is an index of DOM aromaticity^43^. Fluorescence index (FI) was determined as the ratio of 470 and 520 nm fluorescence intensities at 370 nm excitation and FI used to identify sources of DOM (terrestrial vs microbial)^44^. Humification index (HIX) was determined as the ratio between area under the emission spectra 435–480 nm and the sum of the peak area 330–345 nm and 435–490 nm and describes the degree of humification of DOM^45^. Spectral slope (*S*) was determined using a non-linear fit of an exponential function to the log transformed absorption spectrum in the ranges of 275–295 nm and 350–400 nm^46^. Slope ratio (*S*_*R*_) was determined as the ratio of the slope (275–295 nm) and slope (350–400 nm) which have been negatively correlated to DOM molecular weight and aromaticity^46^.

Excitation emission matrices (EEM) were conducted on room temperature water at excitations ranging from 240 to 450 nm at 1 nm intervals and emission ranging from 350 to 550 nm at 2.5 nm intervals using a Jobin Yvon Horiba Fluoromax-3 fluorometer. For 2016 samples EEM emission range was extended from 300 to 600 nm, but for purposes of Parallel Factor Analysis (PARAFAC) modeling these were clipped to match samples from previous years.

Parallel Factor Analysis (PARAFAC) was performed on 132 samples of stream water and soil pore water collected in 2011, 2013, and 2016. PARAFAC is a multivariate technique that decomposes fluorescence spectra into individual fluorescence components^47^. Modeling of PARAFAC components followed established procedures and was normalized to reduce collinearity with reversal of the normalization prior to exporting the model^48^. Prior to PARAFAC modeling, data in the region of first-order and second-order scatter was removed.

### FT-ICR MS analysis

Samples analyzed for FT-ICR MS were collected in June 2016 and July 2018 only. DOM was obtained for FT-ICR MS by a solid phase extraction (SPE) method^49^. Briefly, each sample was acidified to pH 2 and passed through a Bond Elute PPL (Agilent Technologies) cartridge. Residual salts were rinsed from the cartridge with acidified (pH 2) Milli-Q water. The stationary phase was then dried with a stream of N_2_ and the DOM was eluted with 100% MeOH (JT Baker) to a final concentration of 50 µg/mL C and stored at 4 °C prior to analysis. Negatively charged ions were produced via direct infusion electrospray ionization at a flow rate of 700 nL/min and analyzed with a custom-built FT-ICR MS equipped with a 21-tesla superconducting magnet^50^.

Each signal>6σ RMS baseline noise was assigned a molecular formula with EnviroOrg©™ software^51^ developed at the NHMFL. The mass measurement accuracy did not exceed 200 ppb. Each molecular formula was classified based on stoichiometry; condensed aromatic (modified aromaticity index (AI_mod_) ≥ 0.67), polyphenolic (0.67> AI_mod_ > 0.5), unsaturated, low oxygen (AI_mod_ < 0.5, H/C < 1.5, O/C < 0.5), unsaturated, high oxygen (AI_mod_ < 0.5, H/C < 1.5, O/C ≥ 0.5), aliphatic (H/C ≥ 1.5, N = 0), peptide-like (H/C ≥ 1.5, N > 0)^52,53^. Although FT-ICR MS allows for the precise assignment of molecular formulae to peaks that may represent multiple isomers, they describe the underlying molecular compounds comprising DOM, therefore the term compound may be used when describing the peaks detected by FT-ICR MS. Signal magnitude was converted to relative abundances by dividing the signal magnitude of an individual peak by the sum of all assigned signals. Subsequently, the percent contribution of each compound category was calculated as the percent that the relative abundance in each compound category contributed to the summed abundance of all assigned formulae.

### Statistical analyses

Normality was tested for all parameters using the Shapiro-Wilk normality test (α ≤ 0.05). Differences across parameters that follow the assumptions of normality were determined using a one-way ANOVA and nonparametric Kruskal-Wallis rank sum test for parameters that violate the assumptions of normality. We used simple linear regressions to determine relationships between DIN V_f_ and DIN concentrations, stoichiometric ratios, and DOM compound groups. DIN concentrations and DOC:DIN ratios were log transformed to normalize data. To determine if watershed characteristics were potential predictors of patterns in stream chemistry across the fire chronosequence, we used stepwise multiple regression analysis with backward selection. Watershed characteristics (area, elevation, slope, aspect), season (as freshet period or low discharge), and years since the last fire were loaded as independent variables and DOC, DON, DIN, DOC:DIN, relative abundance of combined aliphatics and N-containing aliphatics, condensed aromatics, and polyphenolics, and slope ratio as the dependent variables. Final models were used as those with lowest AIC scores. For all statistical analyses α=0.05 and were conducted in RStudio (version 1.2.1335, RStudio, Inc. Team, Boston, MA 2016) using the stat, rcompanion, and MASS packages. The PARAFAC model was validated using split-half analysis using the drEEM toolbox^48^ in MATLAB (MATLAB R2015b; The Mathworks, Natick, USA, 2008).

## Supplementary information

Supplementary Dataset 1.

## Data Availability

Data can be found online at: 10.4211/hs.5af38c0096014e3c96adecb92bc6e26d.

## References

[1] Zimov SA (2006). Permafrost carbon: Stock and decomposability of a globally significant carbon pool. Geophys. Res. Lett..

[2] Schuur EAG (2008). Vulnerability of permafrost carbon to climate change: Implications for the global carbon cycle. Bioscience.

[3] Frey KE, McClelland JW (2009). Impacts of permafrost degradation on arctic river biogeochemistry. Hydrological Processes.

[4] McClelland JW, Stieglitz M, Pan F, Holmes RM, Peterson BJ (2007). Recent changes in nitrate and dissolved organic carbon export from the upper Kuparuk River, North Slope, Alaska. J. Geophys. Res. Biogeosciences.

[5] Kharuk VI, Dvinskaya ML, Petrov IA, Im ST, Ranson KJ (2016). Larch forests of Middle Siberia: long-term trends in fire return intervals. Reg. Environ. Chang..

[6] Shvidenko AZ, Schepaschenko DG (2013). Climate change and wildfires in Russia. Contemp. Probl. Ecol..

[7] Kawahigashi M, Prokushkin A, Sumida H (2011). Effect of fire on solute release from organic horizons under larch forest in Central Siberian permafrost terrain. Geoderma.

[8] Petrone KC, Hinzman LD, Shibata H, Jones JB, Boone RD (2007). The influence of fire and permafrost on sub-arctic stream chemistry during storms. Hydrol. Process..

[9] Betts EF, Jones JB (2009). Impact of wildfire on stream nutrient chemistry and ecosystem metabolism in boreal forest catchments of interior Alaska. Arctic, Antarct. Alp. Res..

[10] Diemer LA, McDowell WH, Wymore AS, Prokushkin AS (2015). Nutrient uptake along a fire gradient in boreal streams of Central Siberia. Freshw. Sci..

[11] Parham LM (2013). Permafrost and fire as regulators of stream chemistry in basins of the Central Siberian Plateau. Biogeochemistry.

[12] Burd, K. *et al*. Seasonal shifts in export of DOC and nutrients from burned and unburned peatland-rich catchments, Northwest Territories, Canada. *Hydrol. Earth Syst. Sci. Discuss*. 1–32 (2018). 10.5194/hess-2018-253

[13] Larouche JR, Abbott BW, Bowden WB, Jones JB (2015). The role of watershed characteristics, permafrost thaw, and wildfire on dissolved organic carbon biodegradability and water chemistry in Arctic headwater streams. Biogeosciences Discuss..

[14] Holmes RM (2012). Seasonal and Annual Fluxes of Nutrients and Organic Matter from Large Rivers to the Arctic Ocean and Surrounding Seas. Estuaries and Coasts.

[15] Prokushkin, A. S., Gleixner, G., McDowell, W. H., Ruehlow, S. & Schulze, E. D. Source- and substrate-specific export of dissolved organic matter from permafrost-dominated forested watershed in central Siberia. *Global Biogeochem. Cycles* **21**, (2007).

[16] Prokushkin AS (2011). Sources and the flux pattern of dissolved carbon in rivers of the Yenisey basin draining the Central Siberian Plateau. Environ. Res. Lett..

[17] Dodds WK (2002). N uptake as a function of concentration in streams. J. North Am. Benthol. Soc..

[18] Mulholland PJ (2009). Nitrate removal in stream ecosystems measured by 15N addition experiments: Denitrification. Limnol. Oceanogr..

[19] Rodríguez-Cardona B, Wymore AS, McDowell WH (2016). DOC:NO3- ratios and NO3- uptake in forested headwater streams. J. Geophys. Res. G Biogeosciences.

[20] Wymore AS, Coble AA, Rodríguez-Cardona B, McDowell WH (2016). Nitrate uptake across biomes and the influence of elemental stoichiometry: A new look at LINX II. Glob. Biogeochem. Cycles.

[21] Newbold JD, Elwood JW, O’Neill RV, Winkle W (1981). Van Measuring nutrient spiralling in streams. Can. J. Fish. Aquat. Sci..

[22] Workshops SS (1990). Concepts and methods for assessing solute dynamics in stream ecosystems concepts and methods for assessing solute dynamics in stream ecosystems. J. North Am. Benthol. Soc..

[23] Peterson BJ (2001). Control of nitrogen export from watersheds by headwater streams. Science (80-.)..

[24] Tank JL, Rosi-Marshall EJ, Baker MA, Hall RO (2008). Are rivers just big streams? A pulse method to quantify nitrogen demand in a large river. Ecology.

[25] Stubbins A (2014). What’s in an EEM? Molecular signatures associated with dissolved organic fluorescence in boreal Canada. Environ. Sci. Technol..

[26] Striegl RG, Aiken GR, Dornblaser MM, Raymond PA, Wickland KP (2005). A decrease in discharge-normalized DOC export by the Yukon River during summer through autumn. Geophys. Res. Lett..

[27] Vitousek PM, Reiners WA (1975). Ecosystem Succession and Nutrient Retention: A Hypothesis. Bioscience.

[28] Holling CS (1973). Resilience and Stability of Ecological Systems. Annu. Rev. Ecol. Syst..

[29] Mroz GD, Jurgensen MF, Harvey AE, Larsen MJ (1980). Effects of Fire on Nitrogen in Forest Floor Horizons. Soil Sci. Soc. Am. J..

[30] Bayley SE, Schindler DW, Beaty KG, Parker BR, Stainton MP (1992). Effects of Multiple Fires on Nutrient Yields from Streams Draining Boreal Forest and Fen Watersheds: Nitrogen and Phosphorus. Can. J. Fish. Aquat. Sci..

[31] Jiang Y (2015). Modeling carbon–nutrient interactions during the early recovery of tundra after fire. Ecol. Appl..

[32] Prokushkin AS, Knorre AA, Kirdyanov AV, Schulze ED (2006). Productivity of mosses and organic matter accumulation in the litter of sphagnum larch forest in the permafrost zone. Russ. J. Ecol..

[33] Prokushkin AS (2018). Permafrost regime affects the nutritional status and productivity of larches in Central Siberia. Forests.

[34] Mann PJ (2012). Controls on the composition and lability of dissolved organic matter in Siberia’s Kolyma River basin. J. Geophys. Res. Biogeosciences.

[35] Wardle DA, Hornberg G, Zackrisson O, Katela-Brundin M, Coomes DA (2003). Long-Term Effects of Wildfire on Ecosystem Properties Across an Island Area Gradient. Science (80-.)..

[36] Wagner S, Jaffé R, Stubbins A (2018). Dissolved black carbon in aquatic ecosystems. Limnol. Oceanogr. Lett..

[37] Ding Y, Yamashita Y, Jones J, Jaffé R (2015). Dissolved black carbon in boreal forest and glacial rivers of central Alaska: assessment of biomass burning versus anthropogenic sources. Biogeochemistry.

[38] Santos F (2019). Fire severity, time since fire, and site-level characteristics influence streamwater chemistry at baseflow conditions in catchments of the Sierra Nevada, California, USA. Fire Ecol..

[39] Ensign SH, Doyle MW (2006). Nutrient spiraling in streams and river networks. J. Geophys. Res. Biogeosciences.

[40] Kellerman AM, Kothawala DN, Dittmar T, Tranvik LJ (2015). Persistence of dissolved organic matter in lakes related to its molecular characteristics. Nat. Geosci..

[41] Ward ND (2013). Degradation of terrestrially derived macromolecules in the Amazon River. Nat. Geosci..

[42] Kilpatrick, F. A. & Cobb, E. D. Techniques of water-resources investigations of the United States Geological Survey:Measurement of discharge. (1985).

[43] Weishaar JL (2003). Evaluation of specific ultraviolet absorbance as an indicator of the chemical composition and reactivity of dissolved organic carbon. Environ. Sci. Technol..

[44] Cory RM, Mcknight DM (2005). Fluorescence Spectroscopy Reveals Ubiquitous Presence of Oxidized and Reduced Quinones in Dissolved Organic Matter Fluorescence Spectroscopy Reveals Ubiquitous Presence of Oxidized and Reduced Quinones in Dissolved Organic Matter. Environ. Sci. Technol..

[45] Ohno T (2002). Fluorescence inner-filtering correction for determining the humification index of dissolved organic matter. Environ. Sci. Technol..

[46] Helms JR (2008). Absorption spectral slopes and slope ratios as indicators of molecular weight, source, and photobleaching of chromophoric dissolved organic matter. Limnol. Oceanogr..

[47] Stedmon, C. A. & Bro, R. Characterizing dissolved organic matter fluorescence with parallel factor analysis: a tutorial. *Limnology and Oceanography: Methods* **6**(11), 572–579 (2008).

[48] Murphy KR, Stedmon CA, Graeber D, Bro R (2013). Fluorescence spectroscopy and multi-way techniques. PARAFAC. Anal. Methods.

[49] Dittmar T, Koch B, Hertkorn N, Kattner G (2008). A simple and efficient method for the solid-phase extraction of dissolved organic matter (SPE-DOM) from seawater. Limnol. Ocean. Methods.

[50] Smith Donald F., Podgorski David C., Rodgers Ryan P., Blakney Greg T., Hendrickson Christopher L. (2018). 21 Tesla FT-ICR Mass Spectrometer for Ultrahigh-Resolution Analysis of Complex Organic Mixtures. Analytical Chemistry.

[51] Corilo, Y. EnviroOrg, Florida State University: Tallahassee. (2015).

[52] Koch BP, Dittmar T (2006). From mass to structure: An aromaticity index for high-resolution mass data of natural organic matter. Rapid Commun. Mass Spectrom..

[53] Spencer RGM (2014). Source and biolability of ancient dissolved organic matter in glacier and lake ecosystems on the tibetan plateau. Geochim. Cosmochim. Acta.

