# Supplementary material for: Wildfires lead to decreased carbon and increased nitrogen concentrations in upland arctic streams
Source: Sci Rep. 2020 May 26;10:8722. doi: 10.1038/s41598-020-65520-0 (PMC7250865; doi:10.1038/s41598-020-65520-0)
Supplement: Supplementary file 1 — Supplementary Dataset 1. [file 41598_2020_65520_MOESM1_ESM.pdf]

Wildfires lead to decreased carbon and increased nitrogen concentrations in upland arctic streams

B. M. Rodríguez-Cardona[1*], A. A. Coble[2], A. S. Wymore[1], R. Kolosov[3], D. C. Podgorski[4], P. Zito[4], R. G. M. Spencer[5], A. S. Prokushkin[3], and W. H. McDowell[1]

Supplementary Table 1. Watersheds sampled in 2011, 2013, 2016, and 2018 with their respective burn year and watershed size. N11 is currently the only site with two recorded burns, in 1899 and 2013.

Site	Year Burned	Watershed area (km^2)	Years sampled
N1	1947	68.7	2013, 2016
N2	1947	254.3	2013, 2016, 2018
N3	1960	67.6	2013, 2016
N4	1960	3.7	2011, 2013
N5	1960	91.68	2016
N6	1960	15.4	2011, 2013, 2016
N7	1960	15.6	2011
N8	1993*	89.1	2011, 2015
N9	1993	20.8	2011, 2013, 2016, 2018
N10	1993	70.5	2011, 2016
N11	1899, 2013	4.9	2013, 2016, 2018
N13	2009	6.9	2013
N14	2009	9.6	2013, 2016
N15	2009*	36	2016
N19	1896	52.2	2016, 2018
N20	1896	3.8	2016, 2018
N10A	1816	40.38	2016

* watersheds that burned ca. 50%

Supplementary Table 2. Mean NH_4^+ and PO_4^{3-} concentrations across the burn gradient for streams sampled June 2013, 2016, and 2018. 10 represent sites burned between 3 and 7 yrs, 25 watersheds burned between 18 and 25 yrs, 50 watersheds between 51 and 57 yrs, 60 watersheds burned between 66 and 71 yrs, and >100 watersheds burned up to 122 years ago. Parentheses represent standard error and superscript represent statistical difference between groups (α=0.05). Respective n-values: NH_4^+ June:16,16,17,4,10, July:5,5,3,10; PO_4^{3-} June:15,15,16,4,10, July:4,5,3,7.

**Years since the last fire**	**NH_4^+ (μg/L)**		**PO_4^{-3} (μg/L)**	
	June	July	June	July
10	5.23 [a] (1.10)	5.35 (1.90)	13.15 [a] (2.19)	7.13 (2.73)
25	7.71 [a] (2.20)	2.93 (0.40)	8.35 [a] (1.42)	4.25 (1.43)
50	8.87 [a] (1.89)	2.78 (0.28)	6.30 [a] (1.09)	2.96 (0.45)
60	11.52 [a] (5.81)	-	11.00 [a] (1.29)	-
>100	10.26 [a] (6.02)	5.98 (1.13)	12.33 [a] (3.66)	9.02 (3.41)
p-value	0.52	0.38	0.12	0.20
df	4	3	4	3

– represents no data available

Supplementary Table 3. Mean DOM optical properties across the burn gradient for streams sampled June 2013, 2016, and 2018. July values are only from 2018, with low n. HIX values are from June 2016 and July 2018 only. 10 represent sites burned between 3 and 7 yrs, 25 watersheds burned between 18 and 25 yrs, 50 watersheds between 51 and 57 yrs, 60 watersheds burned between 66 and 71 yrs, and >100 watersheds burned up to 122 years ago. Parentheses represent standard error and superscript represent statistical difference between groups (α=0.05). Respective n-values: $SUVA_{254}$ June:13,10,13,4,8, July:1,1,1,3; FI June:13,8,13,4,7, July:1,1,1,3; HIX June: 7,6,7,2,3, July: 1,1,1,3; $S_{275\text{-}295}$ June: 11,9,12,4,7, July: 1,1,1,3; $S_{350\text{-}400}$ June:11,9,12,4,7, July: 1,1,1,3; S_R June:11,9,12,4,7, July: 1,1,1,3. Statistical analyses were excluded from July data due to low n.

**Years since the last fire**	**$SUVA_{254}$ ($L\ mg\ C^{-1}\ m^{-1}$)**		**FI**		**HIX**		**$S_{275\text{-}295}$ (nm^{-1})**		**$S_{350\text{-}400}$ (nm^{-1})**		**S_R**	
	June	July	June	July	June	July	June	July	June	July	June	July
10	3.88 [a] (0.11)	2.69	1.31 [a] (0.007)	1.35	0.963 [a] (0.002)	0.82	0.0137 [a] ($1.74x10^{-4}$)	0.0152	0.0173 [a] ($1.15x10^{-4}$)	0.0184	0.79 [a] (0.006)	0.82
25	3.94 [a] (0.10)	2.91	1.29 [a] (0.011)	1.32	0.964 [a] (0.002)	0.86	0.0134 [a] ($2.15x10^{-4}$)	0.0157	0.0173 [a] ($9.96x10^{-5}$)	0.0182	0.78 [ab] (0.010)	0.86
50	4.03 [a] (0.10)	-	1.29 [a] (0.008)	-	0.964 [a] (0.002)	-	0.0132 [a] ($1.50x10^{-4}$)	-	0.0174 [a] ($7.22x10^{-5}$)	-	0.75 [b] (0.005)	-
60	4.44 [a] (0.29)	2.66	1.30 [a] (0.016)	1.34	0.960 [a] (0.000)	0.92	0.0129 [a] ($8.63x10^{-5}$)	0.0170	0.0170 [a] ($5.29x10^{-5}$)	0.0184	0.76 [ab] (0.008)	0.92
>100	3.96 [a] (0.08)	3.45	1.30 [a] (0.012)	1.31	0.964 [a] (0.001)	0.78	0.0131 [a] ($1.59x10^{-4}$)	0.0144	0.0173 [a] ($1.17x10^{-4}$)	0.0189	0.79 [b] (0.007)	0.76
p-value	0.12	-	0.27	-	0.61	-	0.04	-	0.17	-	0.002	-
F-value	1.95	-	-	-	0.67	-	-	-	1.70	-	-	-
df	4	-	-	-	4	-	-	-	4	-	-	-

– represents no data available.

Supplementary Table 4. A four component PARAFAC model for samples from 2016 only.

Component	Excitation	Emission	Description	Literature reference
**1**	<250 (345)	462	UVC humic-like	1a Stedmon and Markager 2005b<br>FH1; Singh et al. 2013
**2**	<250	478	UVA humic-like: expected to be photoresistant & smaller molecular size compounds	C5; Kothawala et al. 2013<br>C1 Ishi and Boyer 2012
**3**	<250 (307.5)	404	UVC humic-like and UVA marine humic-like; expected to be photodegradaded by UVA but not as much as C4 and moderate molecular size between C2 and C4	3; Stedmon and Markager 2005a<br>C3; Ishi and Boyer 2012
**4**	280	510	UVC humic-like and UVA humic-like; expected to be photodegraded by UVA and large molecular size hydrophobic compounds	C3 Kothawala et al. 2013<br>2 Ishi and Boyer 2012

Supplementary Table 5. Summary of the final backward step wise regression model for DOC, DON, and DIN concentrations, DOC:DIN molar ratios, relative abundance of aliphatics (with N-containing aliphatics), condensed aromatics, and polyphenolics, and slope ratio (S_R) as the dependent variables. The initial model for all models included watershed area (km^2), elevation (m), watershed slope, percent of watershed facing north, percent of watershed facing south, years since the last fire (YSF), and season (as categorical variable and grouped as freshet period or low discharge) as independent variables. Beta values represent the coefficients of the final model with the associated p-value in bold for those values that were statistically significant ($\alpha < 0.05$). All final models are statistically significant ($\alpha < 0.05$). – represents variables that were dropped from the final model.

**Dependent Variable**	**Initial Model**		**Final Model**	
	**AIC**	**R^2**	**AIC**	**R^2**
DOC	241.07	0.46	239.14	0.47
DON	-420.25	0.28	-422.36	0.29
DIN	-574.06	0.20	-577.88	0.23
DOC:DIN	1276.35	0.09	1271.74	0.10
Aliphatics	55.51	0.70	49.9	0.72
Condensed Aromatics	-50.88	0.48	-55.62	0.50
Polyphenolics	12.17	0.56	8.76	0.60
S_R	-363.65	0.92	-369.66	0.93

**Independent Variable**	**Area (km^2)**		**Elevation (m)**		**Slope**		**% North**		**% South**		**YSF**		**Season**	
	**β**	**p-value**	**β**	**p-value**	**β**	**p-value**	**β**	**p-value**	**β**	**p-value**	**β**	**p-value**	**β**	**p-value**
DOC	-0.03	**0.02**	0.02	0.17	-	-	0.20	**0.002**	0.14	0.24	0.08	**< 0.01**	4.43	**< 0.01**
DON	-0.0006	0.05	0.0006	0.08	-	-	0.002	0.17	0.003	**0.03**	0.001	**< 0.01**	0.06	**0.007**
DIN	$-1.83x10^{-4}$	0.05	-	-	$1.54x10^{-2}$	**0.0004**	$-1.13x10^{-3}$	**0.047**	$-1.23x1^{-3}$	**0.045**	$-2.24x10^{-4}$	**0.04**	-	-
DOC:DIN	-	-	-	-	-287.05	**0.04**	-	-	-	-	10.81	**0.006**	603.86	0.08
Aliphatics	-	-	-	-	-	-	0.06	0.15	0.08	0.11	-	-	-8.03	**< 0.01**
Condensed Aromatics	-	-	-	-	-	-	0.03	**0.005**	0.05	**0.0003**	0.004	0.09	0.98	**0.0002**
Polyphenolics	0.007	0.06	-	-	-	-	0.04	0.10	0.08	**0.02**	0.009	0.11	2.87	**< 0.01**
S_R	-	-	-	-	-	-	-	-	$-6.96x10^{-4}$	**0.0009**	$-3.46x10^{-4}$	**0.0002**	-0.22	**< 0.01**

Supplementary Table 6. Details on individual nutrient pulse additions per site from 2016 through 2018 which includes additions where NH_4 was added with PO_4, NH_4 only, and NO_3 only but here we only present uptake metrics for NH_4 and NO_3 only. The experimental reach length, average stream width and depth, discharge (Q), added mass of sodium chloride (NaCl), sodium nitrate ($NaNO_3$), and ammonium sulfate ($(NH_4)_2SO_4$), and uptake metrics for each addition as uptake length (S_w), uptake velocity (V_f), areal uptake (U). * represent undetectable uptake, # represent dates where stream depth measurements could not be taken, and – represent that the given solute was not added for that pulse addition.

Site	Date	Added Solutes	Uptake Nutrient	Reach length (m)	Width (m)	Depth (cm)	Q (L/s)	NaCl (kg)	$NaNO_3$ (g)	$(NH_4)_2SO_4$ (g)	S_w (m)	V_f (mm min^{-1})	U ($\mu g\ m^{-2}$ min)
N11	6/5/16	NH_4+PO_4	NH_4	100	2.69	16.78	148.09	3.90	NA	249.6	557.76	5.92	14.81
N11	6/5/16	NO_3	NO_3	100	2.69	16.78	148.09	4.12	17.2	-	*	*	*
N9	6/6/16	NH_4+PO_4	NH_4	190	4.13	34.2	1041.13	10.00	NA	183	501.73	30.15	75.37
N9	6/6/16	NO_3	NO_3	190	4.13	34.2	1041.13	12.00	328	-	2182.55	6.93	21.14
N6	6/7/16	NH4	NH_4	40	3.6	#	494.92	10.00	-	45	384.58	21.45	53.62
N6	6/7/16	NO_3	NO_3	132	3.6	#	494.92	11.00	41	-	201.60	40.92	81.83
N20	6/8/16	NH_4+PO_4	NH_4	100	2.06	18.25	110.4	3.42	-	70.2	386.44	8.32	20.80
N20	6/8/16	NO_3	NO_3	100	2.06	18.25	110.4	6.09	56.1	-	973.30	3.30	23.62
N11	6/11/16	NH_4+PO_4	NH_4	100	1.78	15	25.2	3.02	-	267.6	221.14	3.84	9.60
N11	6/11/16	NO_3	NO_3	100	1.78	15	25.2	3.00	13.6	-	252.61	3.36	393.76
N9	6/12/16	NH_4+PO_4	NH_4	90	3.24	19.16	172	8.40	NA	178.3	*	*	*
N9	6/12/16	NO_3	NO_3	190	3.24	19.16	172	9.04	342.3	-	*	*	*
N6	6/13/16	NH_4+PO_4	NH_4	40	3.85	40	181.17	2.65	NA	17.2	165.68	17.04	42.60
N6	6/13/16	NO_3	NO_3	132	3.85	40	181.17	2.89	7.5	-	*	*	*
N20	6/14/16	NH_4+PO_4	NH_4	100	1.64	17.39	34.07	1.52	NA	20	217.36	5.73	14.34
N20	6/14/16	NO_3	NO_3	100	1.64	17.39	34.07	1.57	18.1	-	*	*	*
N11	6/17/16	NO_3	NO_3	100	1.4	9.97	4.11	0.45	3.9	-	*	*	*
N20	6/19/16	NO_3	NO_3	65	1.06	14.83	8.09	0.62	9.5	-	*	*	*
N11	6/4/17	NH_4+PO_4	NH_4	100	1.66	0.09	29.45	2.00	-	169.7	518.58	2.05	19.58
N11	6/4/17	NO_3	NO_3	100	1.66	0.09	29.45	2.00	73	-	*	*	*
N4	6/6/17	NH_4+PO_4	NH_4	100	1.60	0.17	33.70	2.00		169.7	221.35	5.71	49.42

N4	6/6/17	NO_3	NO_3	100	1.60	0.17	33.70	2.00	72.9	-	*	*	*
N11	6/19/17	NH_4+PO_4	NH_4	100	1.50	0.13	19.16	0.75	-	106.2	222.37	3.45	32.87
N11	6/19/17	NO_3	NO_3	100	1.50	0.13	19.16	0.75	27.3	-	*	*	*
N11	7/19/17	NH_4+PO_4	NH_4	38	0.46	0.09	0.79	0.05	-	6.4	34.69	2.97	35.59
N11	7/19/17	NO_3	NO_3	38	0.46	0.09	0.79	0.05	4.1	-	*	*	*
N9	7/22/17	NH_4+PO_4	NH_4	40	1.43	0.06	3.143	0.06	-	27	17.85	7.38	31.36
N9	7/22/17	NO_3	NO_3	40	1.43	0.06	3.143	0.06	10.9	-	392.50	0.34	2.45
N20	7/23/17	NH_4+PO_4	NH_4	29	0.58	0.04	0.866	0.06	-	12.7	130.48	0.70	1.76
N20	7/23/17	NO_3	NO_3	29	0.58	0.04	0.866	0.06	16.4	-	*	*	*
N11	7/11/18	NO_3	NO_3	40	1.51	#	3.00	0.02	2	-	28.64	4.16	440.96
N11	7/12/18	NH_4	NH_4	30	1.52	#	2.00	0.03	-	0.5	*	*	*
N20	7/15/18	NH_4	NH_4	100	1.69	16.52	51.18	0.38	-	3.8	130.15	14.04	75.39
N20	7/15/18	NO_3	NO_3	100	1.69	16.52	51.18	0.30	12.2	-	350.85	5.21	13.42
N20	7/15/18	NO_3	NO_3	100	1.69	16.52	51.18	0.22	9.5	-	187.63	9.74	24.84
N9	7/16/18	NO_3	NO_3	21.4	1.32	4.29	0.869	0.05	2.8	-	62.05	0.64	19.61
N9	7/17/18	NH_4	NH_4	15	1.48	3.08	0.457	0.05	-	3.4	5.96	3.12	10.64
N2	7/19/18	NO_3	NO_3	77.2	8.07	9.95	37.56	0.23	10.9	-	27.66	10.11	129.14
N2	7/20/18	NH_4	NH_4	77.2	8.57	9.69	34.15	0.41	-	77.5	30.21	7.92	26.55
N19	7/24/18	NH_4	NH_4	60	2.45	14.86	19.94	0.40	-	3.8	161.69	3.02	7.55
N19	7/24/18	NO_3	NO_3	60	2.45	14.86	18.35	0.40	16.5	-	*	*	*
N20	7/25/18	NH_4	NH_4	86.5	1.54	12.11	13.97	0.20	-	1.9	70.10	7.33	16.13
N20	7/29/18	NO_3	NO_3	86.5	1.59	10.79	9.48	0.20	8.7	-	*	*	*
